# Exstrophie vésicale : à propos d'un cas diagnostiqué tardivement

**DOI:** 10.11604/pamj.2014.17.172.3031

**Published:** 2014-03-07

**Authors:** Michel Tshimbayi, Danny Ndua, Costa Kazadi, Laurent Shamashanga Kwete, Marcellin Bugeme, Patrick Kiopine Mubinda, Olivier Mukuku

**Affiliations:** 1 Faculté de Médecine, Université de Lubumbashi, République Démocratique du Congo; 2 Faculté de Pharmacie, Université de Lubumbashi, République Démocratique du Congo

**Keywords:** Extrophie vésicale, malformation congénitale, Lubumbashi, RD Congo, bladder exstrophy, congenital malformation, Lubumbashi, Congo DR

## Abstract

L'exstrophie vésicale est une forme particulière de malfaçon du tractus génito-urinaire. Son diagnostic est possible par l’échographie dès le premier trimestre de grossesse mais dans la plupart des pays en développement il est diagnostiqué à la naissance faute par manque de surveillance prénatale. Nous rapportons un cas que nous a été amené pour prise en charge d'une plaie hypogastrique depuis la naissance et après une exstrophie vésicale fut diagnostiquée.

## Introduction

Dans les sociétés africaines, l´accouchement d´un enfant malformé est vécu comme un véritable drame compte tenu d´une part des considérations mystico-religieuses qui l´entourent et d´autre part, du poids qu´elle constitue pour les familles. L'exstrophie vésicale est une forme particulière de malfaçon du tractus génito-urinaire; elle est rare et sa fréquence est estimée à un cas sur 10000 à 50000 naissances [1]. Nous rapportons un cas que nous a été amené pour prise en charge d'une plaie hypogastrique depuis la naissance et après une exstrophie vésicale fut diagnostiquée.

## Patient et observation

**Figure 1:**
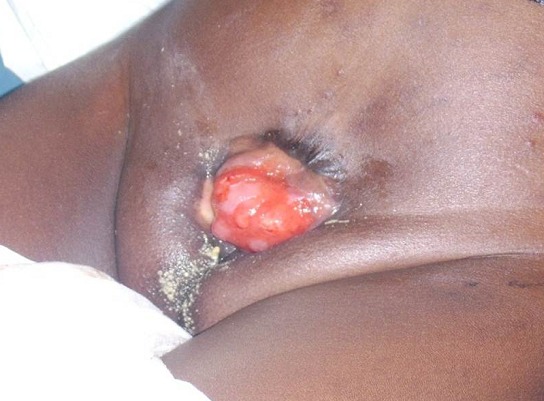
Fillette de 5 ans présentant une exstrophie vésicale (vue

**Figure 2:**
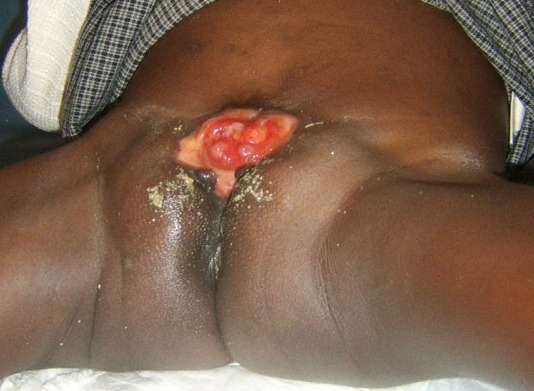
Fillette de 5 ans présentant une exstrophie vésicale (vue inférieure)

Il s'agit d'une fillette âgée de 5 ans qui a été amenée par ses parents pour masse rougeâtre hypogastrique laissant couler des urines. Cette masse était prise pour une plaie (congénitale) par les parents qui y appliquaient des produits traditionnels pour soigner ce qu'ils considéraient comme une plaie. Elle était née à domicile à terme et eutociquement. Dans ses antécédents prénataux, sa mère signale qu'aucune surveillance prénatale n'avait faite et était sujette d'infections génito-urinaires à répétition non traitées. Ses parents sont analphabètes et habitent dans un village à une dizaine de kilomètres de la ville de Lubumbashi. Aucune notion de consanguinité parentale n'avait été notée. Jusqu’à cet âge, la patiente n'avait reçu aucun vaccin et n'avait été examiné par aucun médecin. L'examen clinique à l'admission relève un bon état général. Au niveau de l'hypogastre, nous avons noté la présence d'une structure rougeâtre, ovoïde d'environ 7 centimètres de petit axe et 10 centimètres de grand axe laissant couler les urines dans sa partie supérieure à ses deux extrémités (Figure 1). L'examen des organes génitaux note une vulve est incomplète, un clitoris est bipartite (deux hémiclitoris), des grandes et petites lèvres non identifiables et la présence d'une petite empreinte faisant office d'orifice vaginale (Figure 2). La patiente présentait une démarche caractéristique dite « en canard ». L’échographie abdomino-pelvienne montre un utérus de dimensions 14x7 mm en postérieur de la structure rougeâtre mais la vessie n'est pas identifiée. L'urographie intraveineuse a montré un rein droit fonctionnel et morphologiquement normal et un rein gauche fonctionnel mais avec duplicité pyélo-calicielle, des uretères mis en évidence jusqu'au pelvis, la vessie était non identifiée; le produit de contraste opacifie les linges de protection. Les examens de laboratoire (sanguin et urinaire) réalisés étaient dans les normes. Par manque d’équipement adéquat pour sa prise en charge, la patiente fut transférée en dehors du pays.

## Discussion

L'exstrophie vésicale résulte d'une anomalie de la membrane cloacale qui forme la paroi abdominale infra-ombilicale pendant les premières semaines de la vie embryonnaire [2] et son diagnostic repose sur une non-visualisation de la vessie théoriquement possible dès l’échographie du premier trimestre mais est, dans la plupart des cas, affirmé seulement à l’échographie morphologique du deuxième trimestre [3]. Une fois diagnostiquée, l'extrophie vésicale constitue une cause d'interruption médicale de la grossesse [3]. Cette malformation a d'importantes conséquences esthétiques et fonctionnelles et demande une prise en charge multidisciplinaire spécialisée en vue d'une information éclairée aux parents. La poursuite de la grossesse est actuellement envisageable [3] car certains patients porteurs de cette malformation atteignent l’âge adulte [1]. Son évolution spontanée est dominée par deux risques: l'altération progressive des uretères puis des reins par infection ascendante et sténose, mais aussi et surtout la cancérisation qui est 200 fois supérieure à la normale. Si le diagnostic est posé en période post natale, le traitement de cette pathologie consiste actuellement en une fermeture de la plaque permettant une reconstruction pariétale parfaite. Mais étant donné que l'exstrophie vésicale est considérée comme une semi-urgence néonatale et sa fermeture réalisée très tôt dans les 48 à 72 heures qui suivent la naissance pour espérer 80% de succès [4]. Il s'agit d'une chirurgie lourde et complexe [5] qui, jusqu’à ces jours, dans notre milieu, il est vraiment difficile voire impossible d'en bénéficier par manque d’équipements nécessaires. D'où l'importance de sensibiliser les femmes enceintes dans les pays à faible pénétration sanitaire comme la République Démocratique du Congo à suivre les consultations prénatales car un diagnostic prénatal permettrait d'envisager l'option d'interrompre médicalement la grossesse ou de la poursuivre avec option d'une possible prise en charge en dehors du pays.

## Conclusion

Le fait du manque de suivi des consultations prénatales au cours de la grossesse explique le diagnostic de plusieurs malformations dont l'extrophie vésicale en période postnatale dans notre milieu où sa prise en charge est pratiquement impossible.
